# Human mesenchymal stem cell treatment of premature ovarian failure: new challenges and opportunities

**DOI:** 10.1186/s13287-021-02212-0

**Published:** 2021-03-03

**Authors:** Yun-Xing Fu, Jing Ji, Fang Shan, Jialing Li, Rong Hu

**Affiliations:** 1 Ningxia Medical University, General Hospital of Ningxia Medical University, Yinchuan, 750004 Ningxia China; 2 Reproductive Medicine Center, General Hospital of Ningxia Medical University, Key Laboratory of Fertility Preservation and Maintenance of Ministry of Education, Ningxia Medical University, Yinchuan, 750004 Ningxia China

**Keywords:** Mesenchymal stem cells, Ovarian function, Premature ovarian failure

## Abstract

Premature ovarian failure (POF) is one of the common disorders found in women leading to 1% female infertility. Clinical features of POF are hypoestrogenism or estrogen deficiency, increased gonadotropin level, and, most importantly, amenorrhea. With the development of regenerative medicine, human mesenchymal stem cell (hMSC) therapy brings new prospects for POF. This study aimed to describe the types of MSCs currently available for POF therapy, their biological characteristics, and their mechanism of action. It reviewed the latest findings on POF to provide the theoretical basis for further investigation and clinical therapy.

## Introduction

Premature ovarian failure (POF) is a common endocrine disease causing female infertility. It is characterized by high gonadotropin expression [follicle-stimulating hormone (FSH) ≥ 40 mIU/mL], low estradiol (E2) expression, and follicular dysplasia in women aged less than 40 years [1]. During 2007–2008, the term primary ovarian insufficiency (POI) was suggested to represent this dysfunction related to very early aging of the ovaries [2]. Causes of POF/POI are unknown and related to many complicated factors such as genetic defects [3], autoimmunity [4, 5], chemotherapy injury [6], and others; POF/POI can present as idiopathic [7]. Currently, the prevention and treatment of POF are extremely limited. The most commonly employed hormone replacement therapy cannot effectively recover ovarian function [8]. Thus, the demand for novel and effective therapeutics for POF has increased. Mesenchymal stem cells (MSCs) are highly important in regenerative medicine because of their inherent regenerative properties [9, 10]. Many reports suggested that human mesenchymal stem cell (hMSC) transplantation was a promising treatment for POF [11]. MSCs are multipotent stem cells that are easy to obtain and have poor immunogenicity [12, 13]. They can be harvested from several adult tissues, such as the bone marrow (BM), umbilical cord (UC), peripheral blood, adipose tissue (AD), placenta, and menstrual fluid [14–20]. They are an excellent source of growth factors/cytokines. In this study, the recently published data on hMSC treatment of POF were summarized, and precise mechanisms underlying the effect of stem cells on POF were explored.

## Present situation in POF

The etiology of POF remains sophisticated, including X-chromosome abnormalities, autosomal genetic abnormalities, autoimmune disorder, and enzymatic defects. The POF induced by chemotherapy drugs is more prominent in young patients [21, 22]. The most common clinical treatment of POF is still hormone replacement therapy (HRT). HRT is indicated to reduce the risk of osteoporosis, cardiovascular diseases, and urogenital atrophy and to improve the quality of life of women with POF. However, the role of HRT in promoting fertility remains controversial. Artificial cycles can never replace natural cycles. HRT is considered unsafe in women with a history of breast cancer or ovarian cancer, and alternative measures should be employed to reduce risks and symptoms associated with POF [23, 24]. The last and the most promising measure resorted to is egg donation for most women with POF. However, egg donation resources are very scarce, and patients receiving donated eggs can never have their biological offspring. Therefore, clinicians are looking for new therapies for POF, and MSC transplantation is a promising treatment.

## Treatment of POF using human MSCs

### Human bone marrow MSCs

#### Characteristics of human bone marrow MSCs

Human bone marrow MSCs (hBMMSCs) have high proliferative potential and the ability to differentiate into adipocytes, chondrocytes, and osteoblasts. hBMMSCs were widely researched for treating tumors [25–27], cartilage repair [28], and myocardial infarction [29]. hBMMSCs are multipotent stem cells that can differentiate into multiple cell types, including osteoclasts, myocytes, macrophages, adipocytes, and cardiomyocytes [30]. hBMMSCs primarily derive their energy from glycolysis. The saturated fatty acid palmitate induces BMMSC apoptosis and decreases proliferation in vivo [31]. Many studies showed that hBMMSCs could regulate cytokine expression in Th1 and Th2 cells [32]. hBMMSCs or extracellular vesicle infusion decreased the expression of pro-inflammatory cytokines and chemokines [33].

#### Effects and mechanisms of hBMMSCs on POF (Fig. 1)

BMMSCs were the first stem cells used to evaluate the therapeutic ability of MSCs against chemotherapy-induced POF rat models [34]. BMMSC therapy was found to be protective against germ cell apoptosis and DNA damage in mice undergoing chemotherapy [35]. Cisplatin-induced granulosa cell (GC) apoptosis was reduced when BMMSCs migrated to GCs in vitro and the antral follicle count, E2 levels, and anti-Müllerian hormone (AMH) levels increased after 30-day BMMSC treatment [36, 37]. The new primordial follicles were formed, and FSH levels were near normal in mice with POF after BMMSC injection [38]. Moreover, BMMSCs can reactivate folliculogenesis [39]. BMMSCs homed in the stroma of injured ovaries and the levels of insulin-like growth factor-1 (IGF-1) and tumor necrosis factor-α (TNF-α) increased in ovaries [40]. Human angiogenin promotes primordial follicle survival and angiogenesis in transplanted human ovarian tissue [41]. The overexpression of miR-21 in BMMSCs could repair the ovarian function in rats with chemotherapy-induced POF, which was related to the inhibition of GC apoptosis by targeting phosphatase and tensin homolog deleted on chromosome ten (PTEN) and recombinant human programmed cell death 4 (PDCD4) [42]. Yang et al. demonstrated that BMMSC-derived exosomes could prevent ovarian follicular atresia in cyclophosphamide (CTX)-treated rats via the delivery of miR-144-5p. Moreover, miR-144-5p was related to the inhibition of GC apoptosis by targeting PTEN [43]. The effect of BMMSCs on POF was positive. However, the treatment efficacy was not sufficient for therapeutic purposes probably due to the loss of transplanted MSCs. Some scholars added simple BMMSC injection to other interventions and found that they had better therapeutic effects. Heat shock pretreatment is an effective method to enhance the anti-apoptotic capacity of BMMSCs and achieve a better treatment effect in POF [44]. In addition, human BMMSC-exosomes resulted in further stimulation of angiogenesis via the Akt/mTOR signaling pathway [45].

**Fig. 1:**
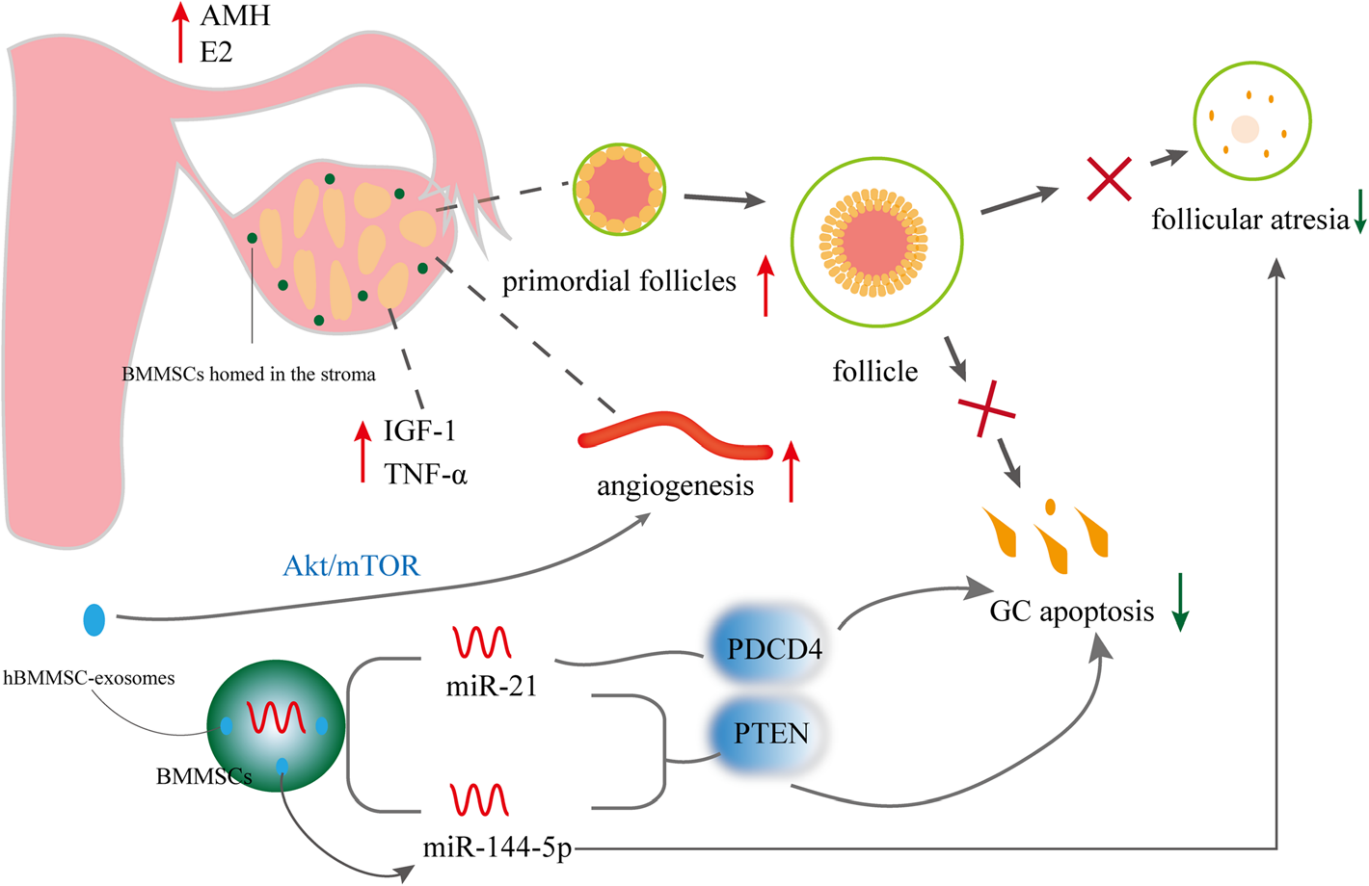
Effects and mechanisms of hBMMSCs on POF

### Human placenta–derived MSCs

#### Characteristics of human placenta–derived MSCs

Human placenta–derived MSCs (hPMSCs) are a subpopulation of villous stromal cells isolated from the full-term placenta. They are widely present in the villous stroma and can differentiate into osteoblasts, cardiomyocytes, smooth muscle cells, adipocytes, endodermic pancreatic islet cells, liver cells, astrocytes, and ectodermic neurons [46–48]. hPMSCs express MSC markers such as stage-specific embryonic antigen (SSEA4), CD44, CD54, CD73, CD90, CD105, CD166, and Oct4 [49]. hPMSCs have immunomodulatory, anti-apoptotic, pro-angiogenic, and anti-fibrotic properties [50–52]. hPMSCs can secrete paracrine factors to exert an antioxidative effect [53] and also secrete pro-angiogenic molecules, including vascular endothelial growth factor (VEGF), basic fibroblast growth factor (bFGF), hepatocyte growth factor (HGF), transforming growth factor-beta (TGF-β), and IGF-1 [54]. HGF secreted by hPMSCs induced lining trophoblast migration in the placental villous microenvironment [49]. Besides, exosomes of hPMSCs also played important biological roles including enhancing angiogenesis in vitro and in vivo [55].

#### Effects and mechanisms of hPMSCs on POF (Fig. 2)

HPMSC transplantation is an effective method to recover ovarian function in mice with CTX-induced POF [56]. A study by Zhang et al. [57] reported reduced levels of FSH, LH, and antizona pellucida antibody (AZP Ab) in serum and increased the levels of AMH and E2 after transplantation of hPMSCs into female BALB/c mice aged 6–8 weeks. hPMSC transplantation significantly recovered the estrus cycle in the POF group and decreased GC apoptosis. The transplantation of hPMSCs suppressed GC apoptosis induced by the endoplasmic reticulum stress inositol-requiring enzyme 1α (IRE1α) signaling pathway in mice with autoimmune-induced POF [58]. In addition, a previous study suggested that hPMSC transplantation reversed ovarian function in mice with POF and decreased the ratios of Th17/Tc17 and Th17/regulatory T (Treg) cells via the phosphatidylinositol 3-kinase/protein kinase B (PI3K/Akt) signaling pathway [59]. The PI3K/Akt signaling pathway is critical for follicular activation, oocyte quality, and GC development [60, 61]. Treg cells are vital in the regulation of autoimmunity and transplantation, producing immunomodulatory cytokines such as transforming growth factor β (TGF-β). Further, TGF-β inhibits the expression of interferon-gamma (IFN-γ). The exceeding IFN-γ levels inhibited ovulation [62]. HPMSC transplantation increased the release of anti-inflammatory cytokines and inhibiting pro-inflammatory production in mice with POF [63]. HPMSCs also inhibited T-cell responses and attenuated inflammation reactions. Epidermal growth factor (EGF) released from hPMSCs improved premature ovarian insufficiency via NRF2/HO-1 activation [64]. The derivation of germ cells, both female and male, was possible in vitro from placenta-derived MSCs [65]. To date, no studies have described the role of hPMSC-derived exosomes (hPMSCs-EXOs) in POF. However, some studies reported that hPMSCs-EXOs increased the levels of anti-inflammatory factor (IL-10) and decreased the levels of other inflammatory factors (IL-1β, IL-8, TNF-α, and NF-κB), thereby reducing the inflammatory response [66].

**Fig. 2:**
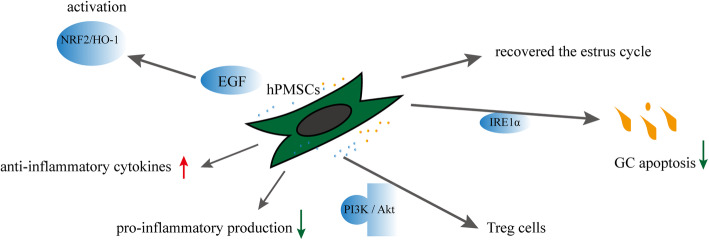
Effects and mechanisms of hPMSCs on POF

### Human amniotic MSCs

#### Characteristics of human amniotic MSCs

Amnion is a waste product of perinatal tissue sources. Therefore, the procedure to obtain human amniotic MSCs (hAMSCs) is noninvasive and represents an advantageous source of cells for cell therapy. hAMSCs are derived from embryonic mesoderm. They are spindle, polygon, or star shaped; the number of long spindle-shaped cells increases significantly after subculture for 3 days [67]. More than 95% of hAMSCs expressed surface markers (CD105, CD29, CD44, CD73, CD49d, and CD90) [68]. Also, cells expressed embryonic/pluripotent stem cell markers such as Oct-4 and SSEA-4 [69]. Some studies identified that hAMSCs secreted a large number of various factors, including EGF, HGF, VEGF, IGF-1, growth differentiation factor 9 (GDF-9), bFGF, and many miRNAs [70]. Compared with hPMSCs, hAMSCs had higher expression of HGF, bFGF, and IGF-1 (>thirtyfold higher) [70]. The expansion potency of hAMSCs was higher than that of adult bone marrow–derived MSCs [17]. hAMSCs had many advantages to be used as a source of allogeneic cells for regenerative medicine, such as high rates of proliferation, self-renewal, multi-differentiation capacity, immunosuppressive and paracrine activity, anti-inflammatory effects, and anti-fibrotic properties [71–76]. hAMSCs might prevent age-related reductions in proliferation and differentiation potentials [77]. hAMSCs that expressed granulocyte chemotactic peptide 2 (GCP-2) and stromal cell–derived factor-1α (SDF-1α) via transcription activator-like effector nuclease (TALEN) enhanced angiogenesis [78]. hAMSCs influenced the mitogen-activated protein kinase signaling pathway to protect cells against oxidative stress–mediated dysfunction [79]. hAMSCs and their conditioned medium (CM) inhibited T cells, reduced the marker expression of Th1 and Th17 cell populations, promoted regulatory T cells, and reduced the cytotoxicity of natural killer cells [80, 81]. Furthermore, CM from hAMSCs had anti-inflammatory [82], anti-neoplastic [83], and immunomodulatory properties [84] and caused oxidative stress inhibition [85].

#### Effects and mechanisms of hAMSCs on POF (Fig. 3)

The secretion of FIGF-1, HGF, GF2, and VEGF by human AMSCs improves the ovarian function of POF. The follicle numbers were recovered to the normal level during week 4 of hAMSC transplantation in the medium-dose CTX group (70 mg/kg). hAMSCs increased ki67+AMH+ cell numbers in patients with diminished ovarian reserve (DOR) and POF (Table 1) [68]. The number of follicles and oocytes and the level of serum E2 significantly increased, but the serum FSH levels significantly decreased in mice with POF + hAMSCs. Levels of Stra8 and integrin beta-1 were upregulated compared with those in model mice [86]. H_2_O_2_ is an endogenous reactive oxygen species, and strong oxidants can cause DNA damage, inflammation, and cell and tissue injury [87–89]. Liu et al. established mice with POF in which bilateral ovaries were burned with 10% hydrogen peroxide. Results indicated that the ovarian function, levels of FSH and estrogen, and fertility rates were recovered in mice with POF-hAMSC transplantation, and normal newborn mice were produced [67]. In mice with natural ovarian aging, hAMSCs exerted a therapeutic effect on mouse ovarian function by increasing follicle numbers. Furthermore, hAMSCs significantly enhanced the proliferation of GCs, and the co-culture of hGCs with growth factors (EGF and HGF from hAMSCs) stimulated the proliferation rate of GCs and inhibited the apoptotic rate more effectively [90]. The efficacy of low-intensity pulsed ultrasound–pretreated hAMSCs were superior to that of normal hAMSCs [89]. Some studies have shown that the hAMSC-mediated activation of GSK3β/β-catenin signaling was dependent upon the PI3K/Akt signaling pathway [91].

**Fig. 3:**
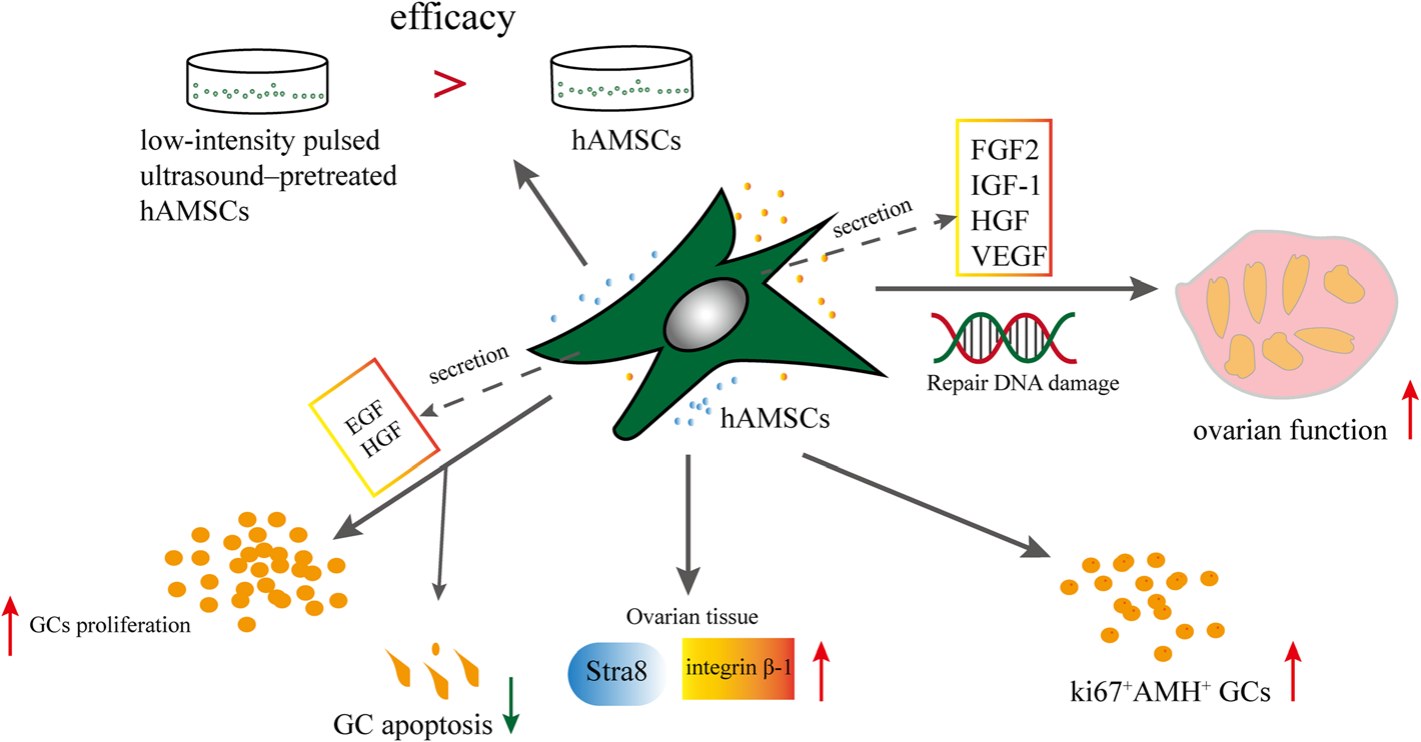
Effects and mechanisms of hAMSCs on POF

**Table 1:** The therapeutic effects of hAMSCs on patients’ GCs with premature ovarian failure [68]

	DOR (*n* = 29)			POF (*n* = 36)		
	Control	hAMSCs	*P* value	Control	hAMSCs	*P* value
No. of AMH cell	22%	83%	< 0.01	4.5%	45%	< 0.01
No. of FSHR cell	41%	84%	< 0.01	17%	51%	< 0.01
No. Of FOXL_2_ cell	34%	88%	< 0.01	19%	70%	< 0.01
No. of CYP19A1 cell	45%	92%	< 0.01	34%	81%	< 0.01

### Human UC–derived MSCs

#### Characteristics of human UC–derived MSCs

MSCs can be isolated from various UC compartments or from the complete UC. In particular, the Wharton’s jelly–derived MSCs (WJ-MSCs) are acquired by removing the UC vessels (arteries and veins). Human UC–derived MSCs (huMSCs) have different biological characteristics, such as painless collection method and infinite self-renewal ability. huMSCs can differentiate into three different germ layers and migrate into the damaged tissue or inflamed regions, contributing to tissue repair. WJ-MSCs have higher proliferation capacity, plasticity, immunomodulatory activity, and self-renewal ability than MSCs from other origins [92]. These huMSCs are suitable candidates for allogeneic transplantation due to their high safety and abundance, shorter expansion times, and low immunogenicity. huMSCs tested positive for surface markers, including CD90, CD73, CD29, CD105, and CD44. They attenuated the inflammatory and oxidative stress, as well as reduced the expression of senescence-related proteins and microRNAs (miRs) [93]. huMSCs exhibited a similar inhibitory effect on T-cell proliferation as on hBMMSCs at a ratio of 1:10, and the percentage of migrating cells was significantly higher in huMSCs compared with BMMSCs [94]. Moreover, no correlation was found between transplanted huMSCs and the risk of tumorigenesis [95].

#### Effects and mechanisms of huMSCs on POF (Table 2)

In 2013, Wang et al. [12] found that umbilical MSCs (uMSCs) could treat POF in mice. The methods for transplanting huMSCs were as follows: intravenous injection (IV) and in situ ovarian microinjection (MI). Both methods of transplantation improved ovarian function, but IV was better able to restore ovarian function compared with MI [96]. Human UC vein MSCs migrated to the cyclophosphamide-injured ovaries, and 57.1%, 32.2%, and 15% were located in the medulla, cortex, and epithelium, respectively [97]. huMSCs promoted the ovarian expression of HGF, VEGF, and IGF-1 and improved ovarian reserve function [98]. The serum levels of P, E2, and IL-4 increased, but the IFN-γ, FSH, and IL-2 levels decreased following huMSC transplantation. Also, the total number of healthy follicles increased and the number of atretic follicles decreased in mice with huMSC-POF [99]. Zheng et al. [100] used cyclophosphamide to create a POF rat model, and cultured huMSCs were transplanted by tail vein injection. They found that huMSCs reduced POF caused by chemotherapy and increased nerve growth factor (NGF) and tropomyosin receptor kinase A (TrkA) levels and decreased follicle-stimulating hormone receptor (FSHR) and caspase-3 levels via the NGF/TrkA signaling pathway. Further, huMSCs improved the ovarian function after paclitaxel injection through a direct triggering effect on the ovarian epithelium and/or indirectly enriching the ovarian niche by regulating the tissue expression of TGF-β, CK 8/18, and PCNA. These molecules are essential in regulating folliculogenesis and inhibiting CASP-3-induced apoptosis [101]. With the advancement of science and technology, scientists have performed in-depth research on the huMSC treatment of POF. huMSC membranous vesicles (MVs) were detectable within the ovaries and migrated to the ovarian follicles 24 h after transplantation. HuMSC-MV transplantation might recover ovarian function by increased angiogenesis through the PI3K/Akt signaling pathway [102]. Exosomes derived from huMSCs (huMSC-EXOs) were also used to prevent and treat chemotherapy-induced GC apoptosis in vitro [103]. In 2020, Yin et al. [104] demonstrated that heme oxygenase-1 (HO-1) expressed in huMSCs was important in restoring the ovarian function, which was mediated via the activation of JNK/Bcl-2 signaling pathway–regulated autophagy and the upregulation of the circulation of CD8^+^CD28− T cells. Collagen scaffold loaded huMSC transplantation in mice with POF, which improved ovarian volume and number of antral follicles and promoted ovarian angiogenesis with the increased expression of CD31; the treatment effect was very significant [105]. huMSCs on a collagen scaffold (collagen/UC-MSCs) can activate primordial follicles by phosphorylation of FOXO3a and FOXO1 in vitro [106].

**Table 2:** Effects and mechanisms of huMSCs on POF

Infertility model	Treatment	Main effect of huMSCs on POF	Species	References
CTX-induced POF	Intravenous injection	Stem cell homing (+), number of healthy follicles at different stages (+), granulosa cell apoptosis (−)	Mice	[12]
Super-ovulation-induced POF	Intravenous injection (IV) and situ ovarian micro injection (MI)	Restore ovarian function (+, IV > MI) Homing in the medulla, cortex, and epithelium of injured ovaries	Mice	[96, 97]
Aging female rats	IV	HGF, VEGF, and IGF-1 (+)	Rats	[98]
ZP3-induced POF	IV	E2, P, and IL-4 (+); FSH, IFN-γ, and IL-2(−); Th1/Th2 (−); improve endometrial conditions (+); number of healthy follicles (+); number of atretic follicles (−)	Mice	[99]
CTX-induced POF	IV	Folliculogenesis (+), NGF and pregnancy rate (+), NGF and TrkA (+), FSHR and caspase-3 (−)	Rats	[100]
Paclitaxel-induced POF		FSH (−); E2 (+); antral follicle (+); CK 8/18, TGF-ß, and PCNA (+); CASP-3(−)	Rats	[101]
Busulfan and CTX-induced POF	HuMSC-MVs (IV)	Homing (+); ovarian weight (+); ovarian angiogenesis (+); recover the disturbed estrous cycle (+); total AKT, p-AKT, VEGF, IGF, and angiogenin (+)	Mice	[102]
Cisplatin-induced POF	HuMSC-EXOs	Granulosa cell apoptosis (−), DNA repair proteins (+)	GCs	[103]
Busulfan and CTX-induced POF	Co-culture of UC-MSCs and GCs (i.p.)	HO-1 expressed in UC-MSCs can restore the ovarian function JNK/Bcl-2-associated cytokines (+)	Mice	[104]
CTX-induced POF	Collagen/UC-MSC transplantation	Ovarian volume (+), number of antral follicles (+), GC proliferation (+), CD31 (+), phosphorylation of FOXO3a and FOXO1 (+)	Mice	[105, 106]

### Human amniotic fluid MSCs (hAFMSCs)

#### Characteristics of hAFMSCs

Amniotic fluid (AF) is routinely obtained via trans-abdominal amniocentesis and contains fetal-derived differentiated and undifferentiated progenitor cells including MSCs. The AF can be obtained by amniotic cavity puncture, and the operation has a minimal effect on maternal and fetal tissues. Currently, hAFMSCs are used for routine prenatal genetic diagnosis [107]. The procedure to obtain hAFMSCs is noninvasive, safe, and without social controversy, consistent with human menstrual blood MSCs [108–110]. In vitro, they can be expanded in different media formulations and exhibit a heterogeneous morphology with obvious epithelioid and fibroblast-shaped cells [111]. hAFMSCs have a high renewal ability and can be extended for more than 250 doublings without any loss of chromosomal telomere length [112]. Furthermore, the potential of induced pluripotent stem cells (iPSCs) derived from AF was better and more effective than that of BMMSCs [113]. AFMSCs expressed pluripotency markers, including Nanog, Oct-4, and sex-determining region y-Box 2 (SOX-2) and SOX-4, and embryonic stem cell markers, including CD117, SSEA-4, TRA1–60, and TRA-1–81 [114, 115]. Some studies reported the presence of common features between primordial germ cells and AFMSCs [116]. AFMSCs are amenable for clinical application and tissue engineering due to their low immunogenicity, anti-inflammatory properties, high proliferative ability, and differentiation capacity in vitro. Also, AFMSCs lacked carcinogenesis after transplantation in nude mice.

#### Effects and mechanisms of hAFMSCs on POF (Fig. 4)

Some studies showed that hAFMSCs expressed multiple growth factors such as EGF, transforming growth factor beta (TGF-β), transforming growth factor alpha (TGF-α), and bone morphogenetic protein 4 (BMP-4) [117, 118]. In 2012, Liu et al. [119] proved that CD44^+^/CD105^+^ hAFMSCs transplanted into the ovaries of mice with POF survived for at least 3 weeks, and CD44^+^/CD105^+^ hAFMSCs underwent normal cycles of cell proliferation and self-renewal in ovarian tissues of mice with POF. HAFMSC-derived exosomes (hAFMSCs-EXOs) enhanced follicular regeneration, regular estrous cycles, and AMH level through the miRNA 21/PTEN/caspase 3 signaling pathway [120]. Compared with hBMMSCs, hAFMSCs secreted higher levels of exosomes. Therefore, hAFMSCs appeared to be a preferable source of exosomes for clinical applications [121]. However, hAFMSCs-EXOs has rarely been reported in studies of POF treatment, so in the future, scholars can focus on the therapeutic effects and mechanism of hAFMSCs-EXOs in POF.

**Fig. 4:**
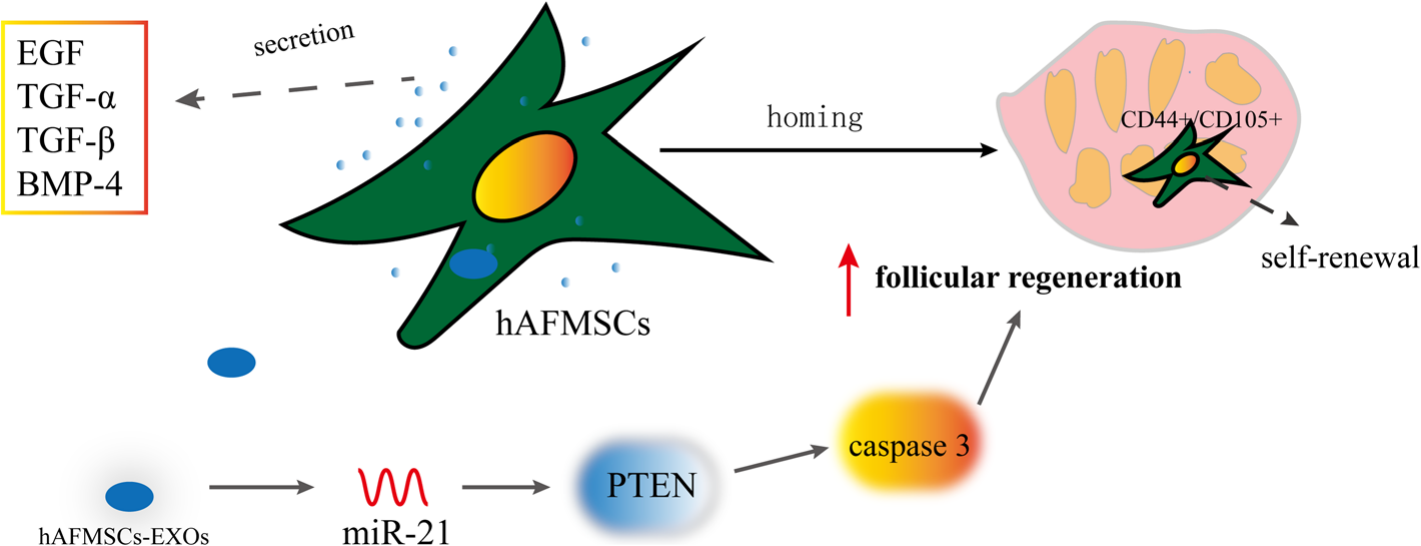
Effects and mechanisms of hAFMSCs on POF

### Human menstrual blood–derived MSCs

#### Characteristics of human menstrual blood–derived MSCs

Human menstrual blood–derived MSCs (hMB-MSCs) were discovered by Meng et al. and Cui et al., as a novel source of MSCs [19, 122]. hMB-MSCs expressed surface markers CD9, CD29, CD41a, CD44, CD59, CD73, CD90, and CD105; human telomerase reverse transcriptase (hTERT); embryonic stem cell marker OCT-4 [19]; and germ cell–specific genes, including DAZL, VASA, c-MOS (oocyte maturation factor mos), STRA8, BLIMP1 (B-lymphocyte–induced maturation protein 1), STELLA (developmental pluripotency-associated protein 3, stella-related protein), SCP3, and SCP1; GDF9, ZPA, and ZPC (zona pellucida gene family) [123]. In addition, hMB-MSCs secrete cytokine growth factors (GM-CSF and PDGF-BB), angiogenic factors (ANG-2, VEGF, HGF, and EGF), and metalloproteinases (e.g., MMP-3 and MMP-10) [19]. hMB-MSCs can differentiate into endothelial, neurocytic, cardiomyocytic, myocytic, cartilaginous, respiratory epithelial, pancreatic, adipocytic, hepatic, and osteogenic cells [124, 125]. Liu et al. demonstrated that hMB-MSCs could differentiate into ovarian tissue–like cells [126], and Lai et al. confirmed the differentiation of hMB-MSCs into germ cells [123]. hMB-MSCs were easy to access compared with hPMSCs and huMSCs. Also, they exhibited MSC-like properties. hMB-MSCs are therefore a novel source of stem cells that can be used for tissue repair [122]. hMB-MSCs also secrete the C-X-C chemokine receptor type 4 (CXCR4) and the respective receptor for stromal cell–derived factor-1 (SDF-1), which plays an important role in MSC migration [127]. Throughout these years, an increasing number of studies paid attention to hMB-MSCs because they possessed higher proliferation rates and painless procedures [124].

#### Effects and mechanisms of hMB-MSCs on POF (Fig. 5)

In 2014, Liu et al. [126] injected (DiO)-labeled hMB-MSCs with green fluorescence into ovaries of POF model mice and found that hMB-MSCs could survive in POF mouse ovaries for at least 14 days. The levels of AMH, FSHR, inhibin α/β, and Ki67 increased following hMB-MSC transplantation in mice with POF. Even more surprising was that the mRNA expression in mouse ovarian cells after hMB-MSC transplantation was similar to that observed in human ovarian cells, suggesting that hMB-MSCs differentiated into ovarian granulosa–like cells in ovaries of mice with POF following stimulation of the ovarian niche. hMB-MSC transplantation could reduce apoptosis of GCs and the fibrosis of the ovarian interstitium. Especially, transplanted hMB-MSCs directly migrated to the ovarian interstitium to repair ovarian function rather than directly differentiating into oocytes. Meanwhile, hMB-MSCs exerted protective effects on damaged ovaries partially by secreting FGF2 [128]. Yan et al. isolated MB-MSCs from three healthy female volunteers and co-cultured them with hGCs treated with epirubicin. They found that MB-MSCs modulated epirubicin-induced DNA damage repair to GCs by regulating protein expression [129]. hMB-MSCs combined with Bushen Tiaochong recipe improved the ovarian function of epirubicin-induced mice with POF, which might be related to inhibiting the expression of GADD45b and promoting the expression of CyclinB1 and CDC2. This therapy combined with stem cells and other treatments also provided a new research direction in POF treatment [130]. Moreover, DiI-labeled hMB-MSCs were found to be localized in the GC layer of immature follicles. hMB-MSCs improved hormone secretion in rats with POF (e.g., AMH, FSHR, FST, E2, and P4). hMB-MSC transplantation not only changed ovarian ultrastructure but also improved ovarian function [131].

**Fig. 5:**
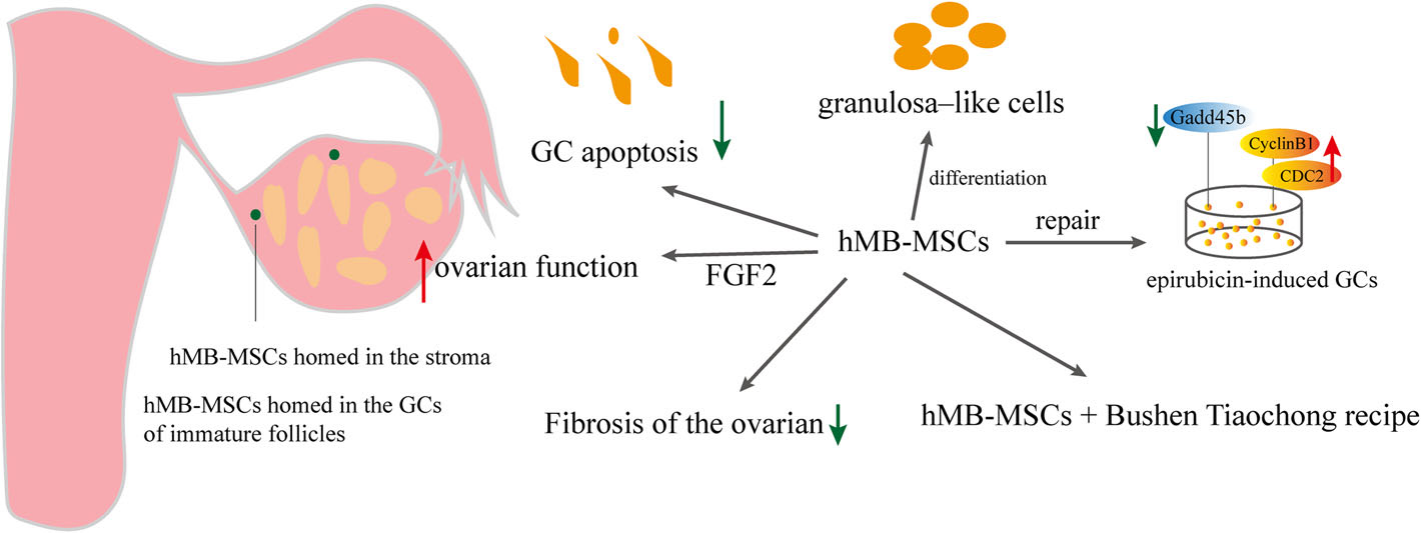
Effects and mechanisms of hMB-MSCs on POF

### Human adipose tissue–derived MSCs (hADMSCs)

#### Characteristics of human adipose tissue–derived MSCs

Adipose tissue is suggested to be an abundant and accessible source of adult MSCs. Human adipose tissue–derived MSCs (hADMSCs) were obtained from the subcutaneous adipose tissue removed during liposuction surgeries or abdominoplasties. Moreover, the high content of hADMSCs in adipose tissue excludes the need for long-term culture in vitro and reduces the risk of chromosomal abnormalities [132]. Compared with hBMMSCs, hADMSCs had higher proliferative and self-renewal abilities and exhibited higher genetic stability in long-term culture [133]. A single unique marker was not identified in hADMSCs, but hADMSCs expressed CD34, CD14, and CD45. hADMSCs inhibited T-cell proliferation by secreting a variety of soluble mediators, including prostaglandin E2 (PGE2) and IFN-γ/indoleamine 2,3-dioxygenase, and inhibiting the NF-κB pathway [134, 135]. Hypoxia-exposed exosomes derived from hADMSCs improved angiogenesis by activating the protein kinase A (PKA) signaling pathway and promoting the expression of VEGF [136].

#### Effects and mechanisms of hADMSCs on POF (Fig. 6)

Human adipose stem cell–derived exosomes (hADMSC-Exos) promoted the hGC proliferation rate and inhibited the hGC apoptotic rate in POI via the regulation of SMAD (SMAD2, SMAD3, and SMAD5) signaling pathway [137]. Combined treatment with hADMSCs and estrogen increased the Treg proliferation and Foxp3 and TGF-β1 mRNA expression in POI and decreased the IFN-γ mRNA expression [138]. Moreover, the combined therapy of ADMSCs, VEGF, and platelet-rich plasma improved rat ovarian function significantly more than expected in the cyclophosphamide-induced POI model. The expression of BMP4, IGF-1, and TGF-β increased in rats with POF [139]. Transplanted hADMSCs were located only in the interstitium of ovaries. hADMSCs secreted FGF2, IGF-1, HGF, and VEGF. hADMSC transplantation improved ovarian function in rats with chemotherapy-induced POI at least partly through a paracrine mechanism [140]. Obviously, the involvement of hADMSCs in the therapeutic mechanism of POF has been less explored. hADMSCs also effectively reduced fibrosis and inflammation [141]. However, whether it affects fibrosis during the treatment of POF still needs further research.

**Fig. 6:**
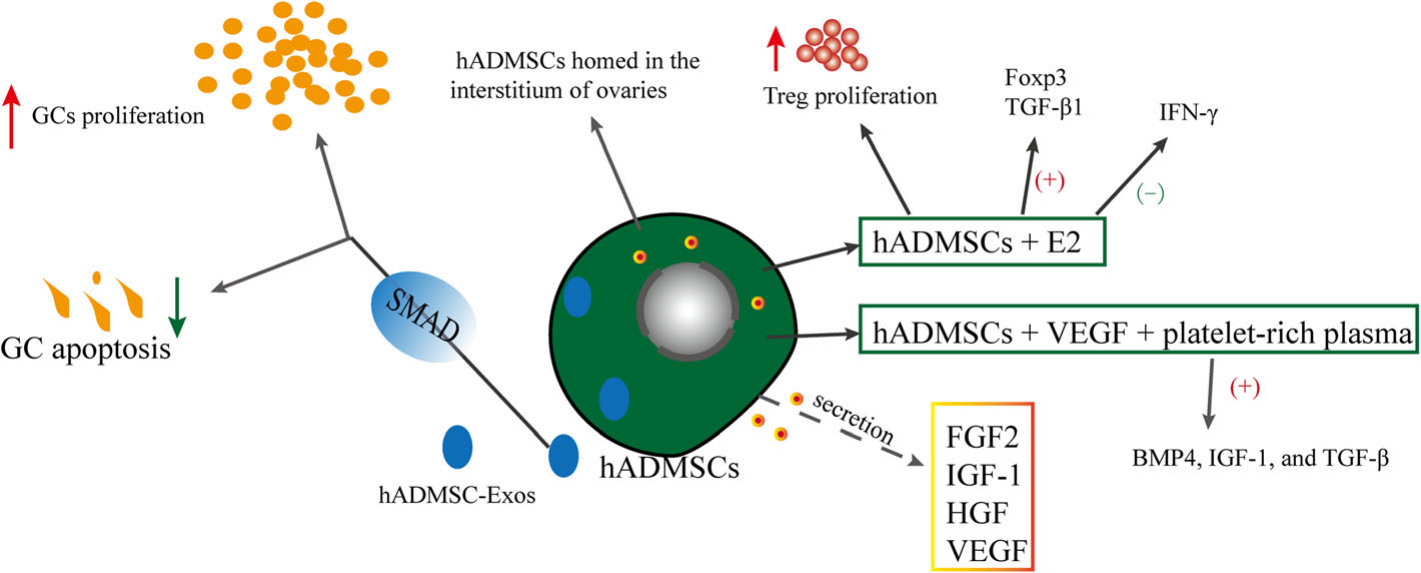
Effects and mechanisms of hADMSCs on POF

### Effects of other MSCs on POF

The peritoneum mesothelial consists of the intraperitoneal or intestinal space, abdominal wall, and omentum. The peritoneum mesothelium lines body cavities and has the same origin as the ovarian surface epithelium. Besides, the repair and reconstruction of peritoneum mesothelial cells can be performed through the secretion of growth factors (bFGF and VEGF), cytokines, and extracellular matrix [142]. Characterized peritoneum MSCs (PeMSCs) were differentiated into ovarian cell–like cells using 10% human follicular fluid and 50% human cumulus-CM for 21 days, and the expression of oocyte (Zp3 and Gdf9), germ cell (Ddx4 amd Dazl), GC (Amh), and theca cell (Lhr) markers was assessed [143]. Skin-derived MSCs (SMSCs) improved the ovarian follicle microenvironment of mice with POF, and the levels of pro-inflammatory cytokines TNF-α, TGF-β, IL-8, IL-6, IL-1β, and IFN-γ significantly decreased [144].

## Problems and prospects

**Fig. 7:**
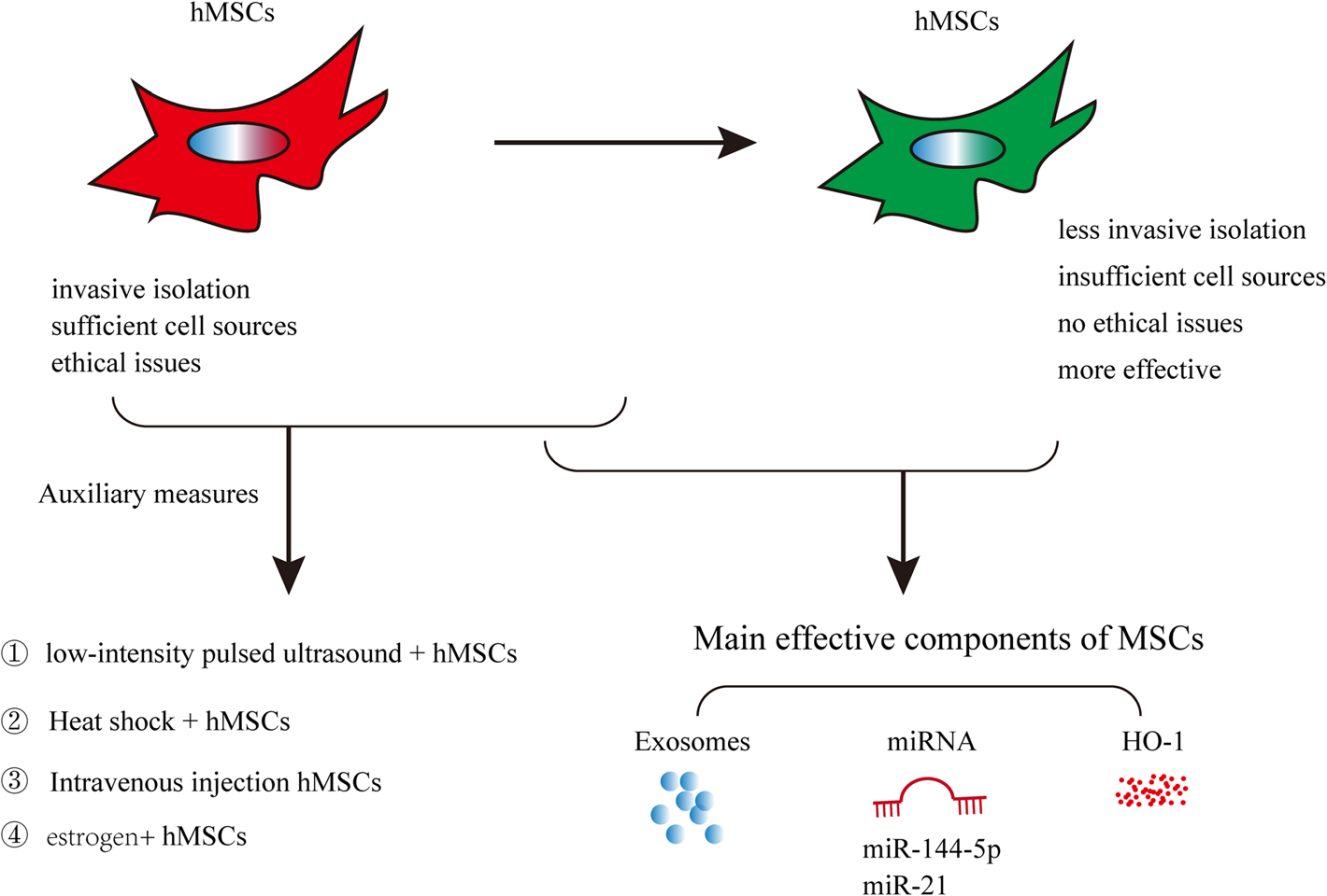
Human mesenchymal stem cell treatment of premature ovarian failure: new challenges and opportunities

The past years have witnessed considerable advances in the knowledge base related to the use of MSCs for regenerative medicine. Recent breakthrough discoveries in engineering MSCs have made them an ideal source for future cell therapy in POF. It is critical to ensure the safety of MSC clinical application by understanding their impacts on tumor initiation and progression. Although many experimental and clinical assays have been developed, clinical applications of MSCs have limitations, including insufficient cell sources, immunogenicity, subculture, and ethical issues. For clinical trials, the functional potential and microbiological safety of cells must be considered, and it should be ensured that cultured cells remain untransformed. Setting up a professional system to test the quality of MSC production is extremely challenging. In addition, the large variability in cell quality is derived from different donors and tissues. Thus, more reliable and effective MSCs obtained using less invasive isolation techniques have become treatment options. Hence, cell-free therapy, which uses stem cells as a source of therapeutic molecules, can be developed to treat different disease models (Fig. 7). The main effective components of MSCs should be identified in the treatment of POF, which can avoid potential side effects caused by unnecessary treatments. Exosomes, which are important messengers between cells, regulate other cells. Numerous studies have been conducted on the biological effects of exosomes secreted by stem cells [145]. On the contrary, MSC-secreted exosomes are smaller and easier to produce and have no risk of tumor formation, facilitating their comprehensive study and clinical use in the future (Fig. 7) [146, 147]. Besides, stem cells can spread rapidly to the nearby organs or tissues, and the collagen scaffolds were believed to support the survival of transplanted cells at the initial phase of transplantation in vivo. For example, transplantation of UC-MSCs on collagen scaffold activates follicles in dormant ovaries of POF patients with long history of infertility. Therefore, MSCs that can be used combined with other therapies to prompt the treatment of POF should be explored.

## Conclusions

The transplantation of MSCs has brought hope for patients with POF. Especially MSCs were obtained using less invasive isolation techniques because they were not only noninvasively obtained but also out of the ethical debate. In addition, cell-free therapy (therapeutic molecules from MSCs) has been extensively researched to overcome such challenges. It is expected that POF can be successfully treated using the main effective components of MSCs and combined therapy soon.

## Data Availability

Not applicable.

## Abbreviations

AD: Adipose tissue
AMH: Anti-Müllerian hormone
BM: Bone marrow
BMP-4: Bone morphogenetic protein 4
BMP-15: Bone morphogenetic protein 15
bFGF: Basic fibroblast growth factor
DOR: Diminished ovarian reserve
E2: Estradiol
ER: Endoplasmic reticulum
EGF: Epidermal growth factor
FSH: Follicle-stimulating hormone
FSHR: Follicle-stimulating hormone receptor
GnRH: Gonadotropin-releasing hormone
GDF-9: Growth differentiation factor 9
GCs: Granulosa cells
GCP-2: Granulocyte chemotactic peptide 2
HO-1: Heme oxygenase-1
HRT: Hormone replacement therapy
hMSCs: Human mesenchymal stem cells
hPMSCs: Human placenta–derived mesenchymal stem cells
hAMSCs: Human amniotic mesenchymal stem cells
hBMMSCs: Human bone marrow mesenchymal stem cells
huMSCs: Human umbilical cord–derived mesenchymal stem cells
hAFMSCs: Human amniotic fluid mesenchymal stem cells
hADMSCs: Human adipose tissue–derived mesenchymal stem cells
HGF: Hepatocyte growth factor
IGF-1: Insulin-like growth factor-1
hTERT: Human telomerase reverse transcriptase
IFN-g: Interferong
IRE1α: Stress inositol-requiring enzyme 1α
LH: Luteinizing hormone
MAP: Mitogen-activated protein kinase
NGF: Nerve growth factor
POF: Premature ovarian failure
POI: Premature ovarian insufficiency
PB: Peripheral blood
PGE2: Prostaglandin E2
PTEN: Phosphatase and tensin homolog deleted on chromosome ten
PDCD4: Recombinant human programmed cell death 4
PeMSCs: Peritoneum mesenchymal stem cells
SDF-1α: Stromal cell–derived factor-1α
SMSCs: Skin-derived mesenchymal stem cells
sSSEA: Tage-specific embryonic antigen
SOX-2: Sex-determining region y-Box 2
TGF-α: Transforming growth factor alpha
TGF-β: Transforming growth factor beta
TNF-α: Tumor necrosis factor-α
TALEN: Transcription activator-like effector nuclease
TrkA: Tropomyosin receptor kinase A
UC: Umbilical cord
VEGF: Vascular endothelial growth factor
